# Augmented reality-guided pelvic osteotomy of Ganz: feasibility in cadavers

**DOI:** 10.1007/s00402-023-05167-4

**Published:** 2023-12-22

**Authors:** Armando Hoch, Florentin Liebmann, Mazda Farshad, Philipp Fürnstahl, Stefan Rahm, Patrick O. Zingg

**Affiliations:** 1 Department of Orthopaedics, Balgrist University Hospital, University of Zurich, Forchstrasse 340, 8008 Zurich, Switzerland; 2 Research in Orthopaedic Computer Science, Balgrist University Hospital, University of Zurich, Zurich, Switzerland

**Keywords:** Augmented reality, Computer assisted surgery, Periacetabular osteotomy, Developmental dysplasia of the hip, Surgical navigation

## Abstract

**Introduction:**

The periacetabular osteotomy is a technically demanding procedure with the goal to improve the osseous containment of the femoral head. The options for controlled execution of the osteotomies and verification of the acetabular reorientation are limited. With the assistance of augmented reality, new possibilities are emerging to guide this intervention. However, the scientific knowledge regarding AR navigation for PAO is sparse.

**Methods:**

In this cadaveric study, we wanted to find out, if the execution of this complex procedure is feasible with AR guidance, quantify the accuracy of the execution of the three-dimensional plan, and find out what has to be done to proceed to real surgery. Therefore, an AR guidance for the PAO was developed and applied on 14 human hip cadavers. The guidance included performance of the four osteotomies and reorientation of the acetabular fragment. The osteotomy starting points, the orientation of the osteotomy planes, as well as the reorientation of the acetabular fragment were compared to the 3D planning.

**Results:**

The mean 3D distance between planned and performed starting points was between 9 and 17 mm. The mean angle between planned and performed osteotomies was between 6° and 7°. The mean reorientation error between the planned and performed rotation of the acetabular fragment was between 2° and 11°.

**Conclusion:**

The planned correction can be achieved with promising accuracy and without serious errors. Further steps for a translation from the cadaver to the patient have been identified and must be addressed in future work.

**Supplementary Information:**

The online version contains supplementary material available at 10.1007/s00402-023-05167-4.

## Introduction

The periacetabular osteotomy (PAO) was introduced by Ganz in 1988 and since then has further developed the possibilities in joint-preserving hip surgery [6]. Its aim is to improve the osseous containment of the femoral head in young adults with dysplasia of the hip (DDH), and some constellations of femoroacetabular impingement or acetabular protrusion [6, 7, 16, 17]. The PAO is technically demanding and only performed by experienced surgeons. Due to limited intraoperative view, complex anatomy and with lack of intraoperative guidance or verification methods for some parts of the procedure, so far, a profound three-dimensional (3D) imagination is crucial. Since in PAO some of the osteotomies are not directly visible and at least by most surgeons, guided by fluoroscopy only, the anatomical understanding significantly exceeds what is typically required in orthopedic surgical procedures.

Incorrectly performed osteotomies or unsatisfactory reorientation of the fragment can lead to serious consequences. Possible results are acetabular fractures and injuries to the acetabular cartilage, whose preservation is the designated goal of the procedure. Furthermore, a fracture of the posterior column can cause instability of the pelvic ring. Intraoperative fractures are reported to occur in 1% of the cases [2]. Both, under- and over-correction, miss the goal of the procedure and leads to an inferior outcome. This complication is also seen in 1% of the cases in the literature, but based on our clinical experience and that of others, a higher rate can be assumed [2]. Moreover, overcorrection or misalignment can provoke a mechanical conflict between the femoral head–neck junction and the acetabular rim resulting in iatrogenic femoroacetabular impingement and the subsequent necessity of a revision procedure [7, 18]. Conversion rate to total hip replacement in these young patients is more than 4% after a mean follow-up of less than 5 years and 38% after 20 years, which is influenced by intraoperative fractures and malcorrection [2, 18].

So far, there have been various approaches to increase the safety and accuracy of this procedure. Today's state-of-the-art technology is intraoperative imaging [3, 12, 13, 21]. Intraoperative fluoroscopy provides the surgeon with feedback on the position of the instruments. The most important aspect is the localization of the chisel when performing the retroacetabular and ischial osteotomies, which cannot directly be visualized without an additional posterior approach. The performed reorientation of the acetabular fragment can be quantified by an intraoperative radiograph, in which certain parameters are measured intraoperatively. According to the measurements, it is then decided whether a subsequent correction is necessary. Nevertheless, intraoperative imaging needs additional personnel and is time and cost consuming. Furthermore, for a young collective, the radiation exposure is not only detrimental [21], but also harmful for medical personnel.

Furthermore, PAO remains an intervention that aims to correct a 3D malorientation. This requires 3D malorientation analysis and 3D correction planning. Such planning cannot be implemented, navigated, and verified intraoperatively with the state-of-the-art 2D guidance. Only 3D technology can build this bridge.

With the recent development in the field of augmented reality (AR), such a 3D technology has become available [4, 8, 11, 14, 15]. In contrast to other surgical guidance methods (patient-specific instruments, optical tracking systems), with AR a procedure can be performed using only optical see-through head-mounted displays (OST-HMD). No change of the standardized surgical procedure, or the need for additional and expensive instruments is necessary. The surgical plan can be made available within the surgical field itself with no necessity for additional monitors. Furthermore, with an OST-HMD, the surgeon is not required to adapt his movements due to line-of-sight issues. Nevertheless, AR-guided interventions are still in their infancy and there is uncertainty about their reliability and accuracy. It has already been demonstrated that PAO can be successfully performed on the sawbone model and satisfactory accuracy can be achieved [11]. In a pilot study on a single cadaver, valuable knowledge regarding technical hurdles was described [9] and the flaws were worked through in a technical publication [1]. We have now carried out a study on cadavers to answer the question if this method is feasible and which steps still need to be optimized before the method can be applied in real surgery.

## Materials and methods

14 hips (7 thawed fresh-frozen human cadavers) were used for this study. The specimen had no history of trauma, malformation, tumor, or surgery. This study was approved by the local ethical committee (KEK Zurich BASEC Nr. 2018-00922). The study was conducted as follows: In the first step, imaging of the cadavers, data processing and planning of the procedure was performed *(Step 1)*. In a second step, the planning was fed into the Microsoft HoloLens 1 (Microsoft Corporation, Redmond, WA, USA) using an in-house developed application for AR-guided navigation and surgery was performed. Regarding this second step, the technical navigation approach *(Step 2-A)* must be differentiated from its practical implementation in the surgical part of the cadaver experiments *(Step 2-B)*. In a last step, post-procedural imaging and outcome measurement was performed *(Step 3)*.

### Step 1: imaging, processing, planning

**Fig. 1:**
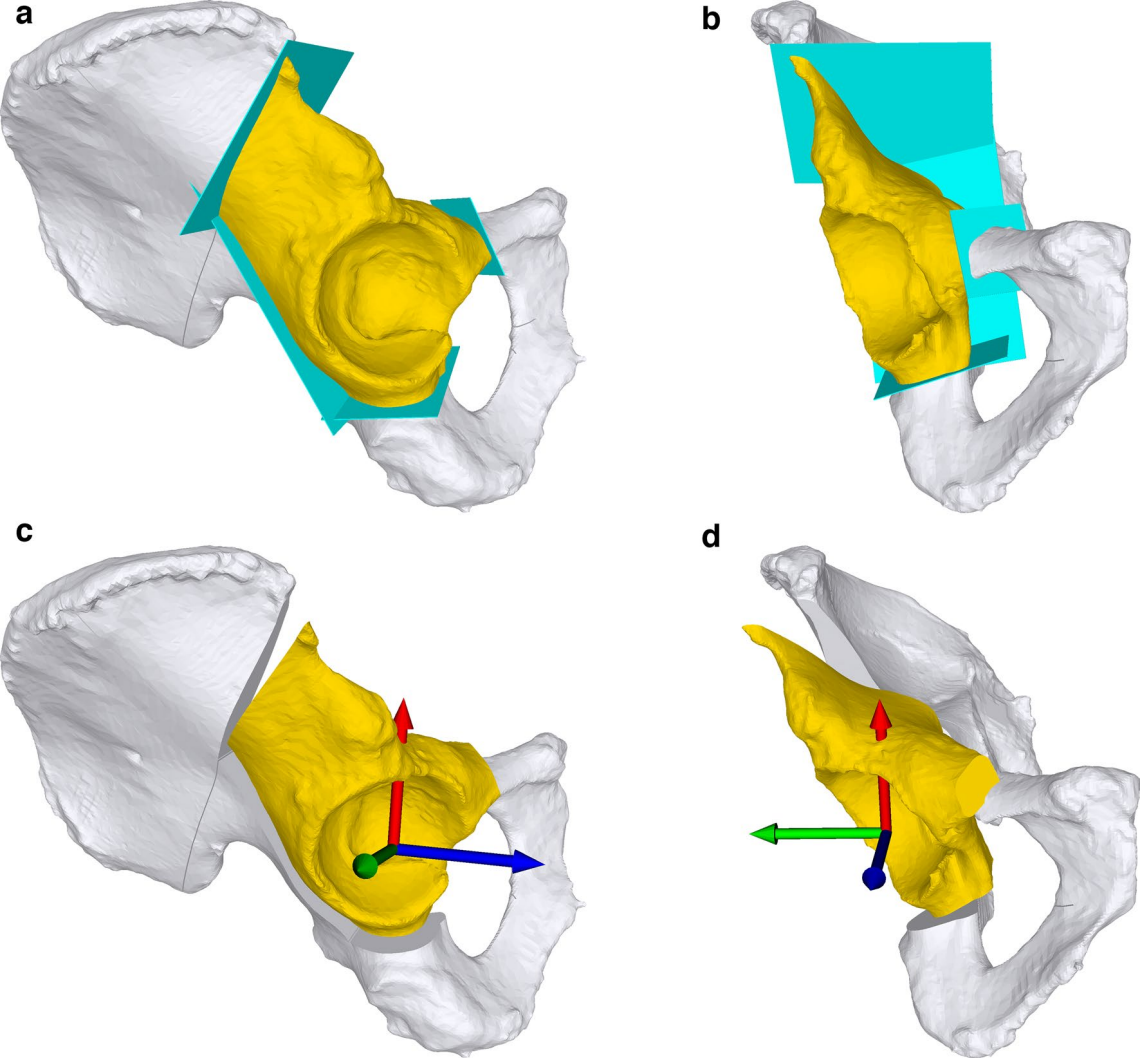
**a** 3D planning of the PAO with cutting planes (turquoise) separating the acetabular fragment (gold) from the remaining pelvis (white) from a lateral view. **b** 3D planning of the PAO with cutting planes (turquoise) separating the acetabular fragment (gold) from the remaining pelvis (white) from an anteroposterior view. **c** 3D planning of reorientation of acetabular fragment (gold) with center of rotation and anatomical planes displayed with a coordinate system (x (red)–y (green)-plane: frontal, x–z(blue)-plane: sagittal) from a lateral view. **d** 3D planning of reorientation of acetabular fragment (gold) with center of rotation and anatomical planes displayed with a coordinate system (x (red)–y (green)-plane: frontal, x–z(blue)-plane: sagittal) from an anteroposterior view

A CT scan of the cadavers was performed using a Somatom Edge CT device (Siemens, Erlangen, Germany). The CT data were segmented using the global thresholding and region growing functionalities of a commercial segmentation software (Mimics Medical, Materialise, Leuven, Belgium) to generate 3D models of the pelvis and femur [5, 10, 19]. The 3D preoperative planning was performed by an experienced hip surgeon (P.O.Z.) using our in-house-developed planning software CASPA (Balgrist University Hospital, Zurich, Switzerland). Therefore, the starting points of the osteotomies and the 3D cutting planes were placed according to Ganz' original description of the periacetabular osteotomy [6] (Fig. 1a and b). However, since this original description leaves some space for interpretation and there are different ways to implement it in three dimensions, a specific 3D option had to be chosen in the planning. As the cadavers had a physiological hip anatomy, the amount and direction of the reorientation of the acetabular fragment were chosen such that they resembled a typical correction in DDH. Since there are no data on this typical three-dimensional correction available so far, we have calculated a mean reorientation value for all planes on the basis of the last 15 PAO carried out in our institution. A rotation of 15° in the frontal plane and 10° in the sagittal plane was applied (Fig. 1c and d). This constant identical correction has a different effect on each 3D planning of the individual cadavers due to the different anatomy.

### Step 2-A: technical navigation approach

This section will explain the hard- and soft-ware components which are used for the surgical navigation described later.

Hardware components:

These are described in Table 1. All components were prepared and laid out in a human cadaver laboratory. The HoloLens was started up and the interpupillary distance of the operator was calibrated using the application delivered by the manufacturer. The pointing device (PD) was equipped with a marker.

**Table 1:** Hardware components

Component	Description	Size [L x W x H, cm]	Producer	Medical Product	Single Use
HoloLens 1	Optical see-through head-mounted display for augmented reality	29 × 22 × 10	Microsoft Corporation, Redmond, WA, USA	No^1^	No
Pointing Device	3D-printed pen-like instrument for touching surfaces, can be equipped with markers	3 × 3 × 10	In-house	Yes	Yes
Mount	3D-printed part with screwhole for fixation to anatomy, can be equipped with marker	3 × 3 × 1	In-house	Yes	Yes
Marker	Sterile QR code-like sticker	2.5 × 2.5 × 0.25	Clear Guide Medical, Baltimore, MD, USA	Yes	Yes
Screws / K-Wires	Surgical implants for temporary fixation of mounts and osteosynthesis of fragment	Various	Synthes, West Chester, PA, USA	Yes	Yes
Surgical Instruments	Surgical instruments as used for periacetabuluar osteotomy in patients	Various	Various	Yes	No

*L* length, *W* width, *H* height, *QR* quick Response

^1^Approved for clinical study

Software components:

Our method was implemented as a holographic Universal Windows Platform application for the Microsoft HoloLens using Unity (Unity Technologies, San Francisco, CA, USA).

The navigation approach required the following major steps: coarse registration between pre- and intraoperative anatomy, fine registration, tracking of pelvis, visualization of osteotomy planes, and tracking of fragment. Our registration approach based on our previous work on radiation-free surface digitization [1, 9] using a custom-made 3D-printed PD. The sterile marker integrated in the PD shows an AprilTag pattern [11, 12] that can be tracked by the two front-facing cameras of the HoloLens using OpenCV [13] and the ArUco library [14, 15].

In this study, a coarse alignment was achieved by capturing three landmarks on the pelvic bone using the PD. Afterwards, the accessible region on the pelvis was digitized into a 3D point cloud by tracking the continuous motion of the PD tip when following the bony anatomy. The anatomical details are described later.

This point cloud was then aligned to the preoperative 3D model using the iterative closest point (ICP) surface registration [16]. Applying osteotomy cuts with a chisel leads to motion of the anatomy and, consequently, to a loss of registration. We addressed this issue by extending the original method with a motion compensation strategy. After successful registration, the pelvis position was stored with respect to the custom-made 3D-printed pelvic mounts equipped with a marker. A description of this strategy can be found in our previous publication[1]. The osteotomies were guided visually by rendering planes for supra- and retroacetabular cuts and chisel positions for the ischial cut, respectively. For the reorientation of the mobilized acetabular fragment, the surgeon could choose between visual guidance where the fragment was rendered at the planned postoperative position and a tracking-based guidance with a display showing the angular and positional deviation between current and targeted fragment position.

### Step 2-B: cadaver experiments, surgical technique

**Fig. 2:**
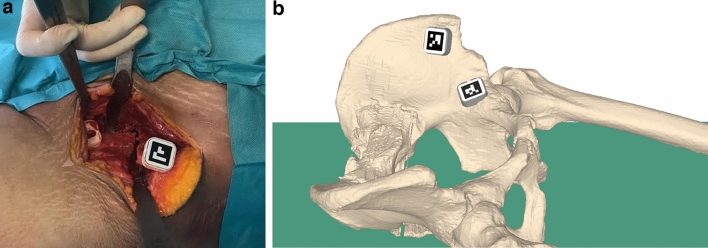
**a** Pelvic mount equipped with marker in intraoperative situs. **b** Schematic model of pelvic and fragment mount position on 3D bone model of a cadaver

**Fig. 3:**
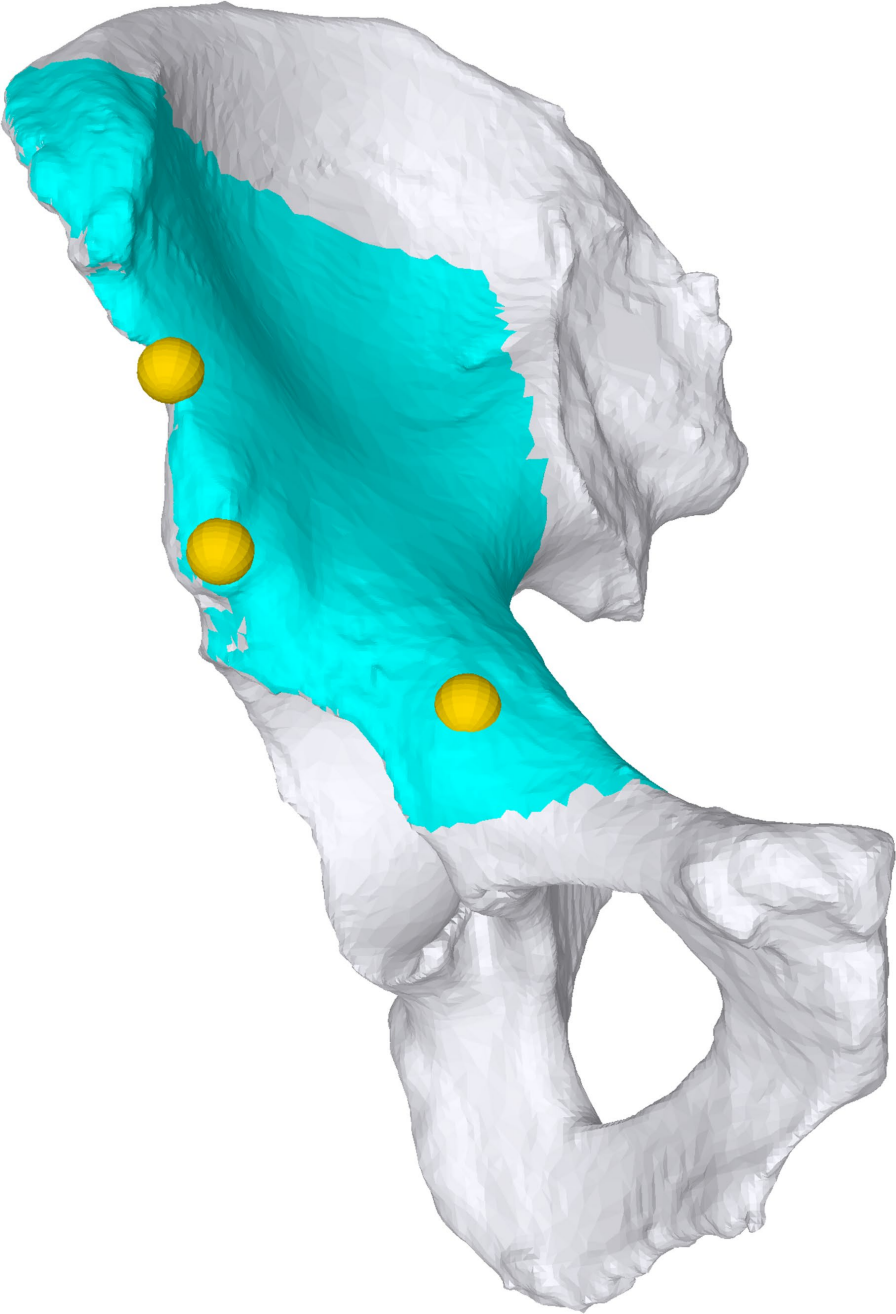
Predefined anatomical landmarks (golden spheres) and accessible surface (blue) for digitization on pelvic bone (white)

**Fig. 4:**
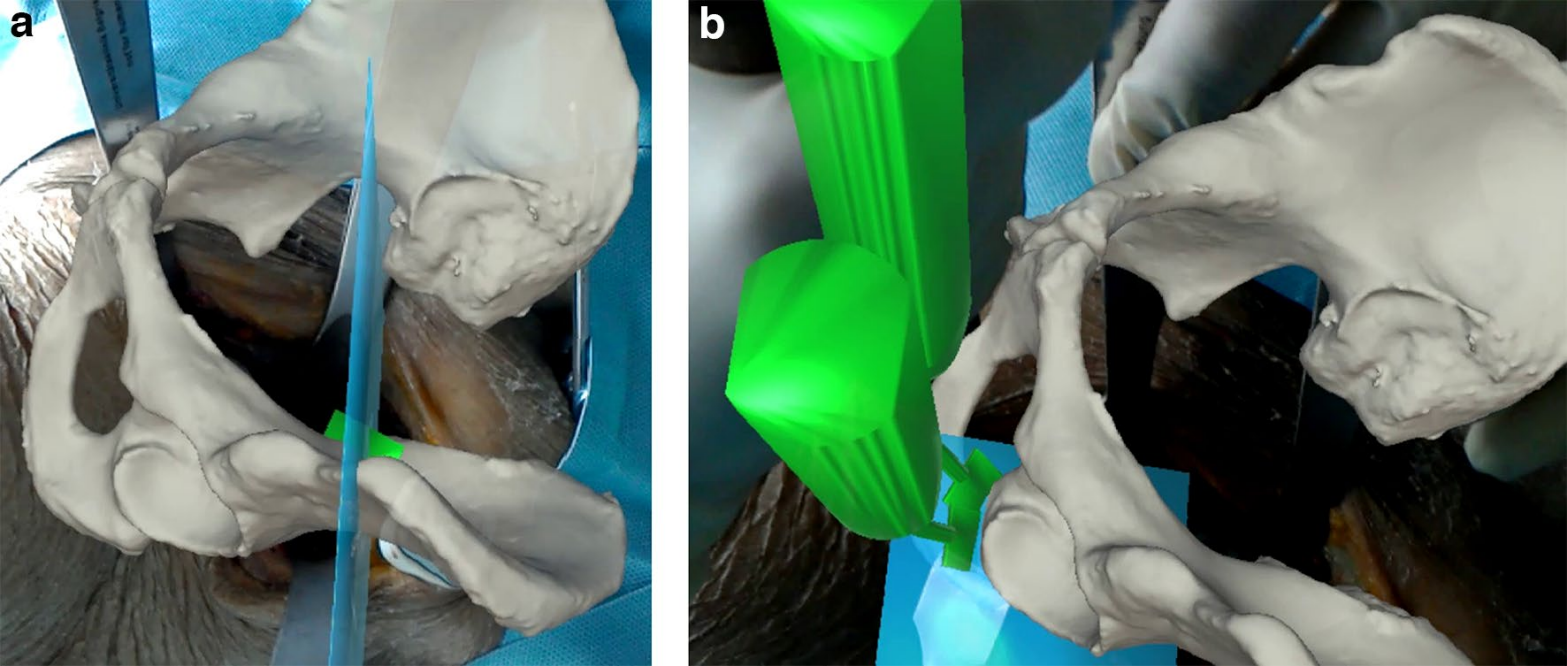
**a** Surgeons intraoperative view with holographic overlay of supraacetabular osteotomy plane (semi-transparent blue) and transition point (green cross). **b** Surgeons intraoperative view with holographic overlay of ischial osteotomy plane (semi-transparent blue) and different chisel positions (green)

**Fig. 5:**
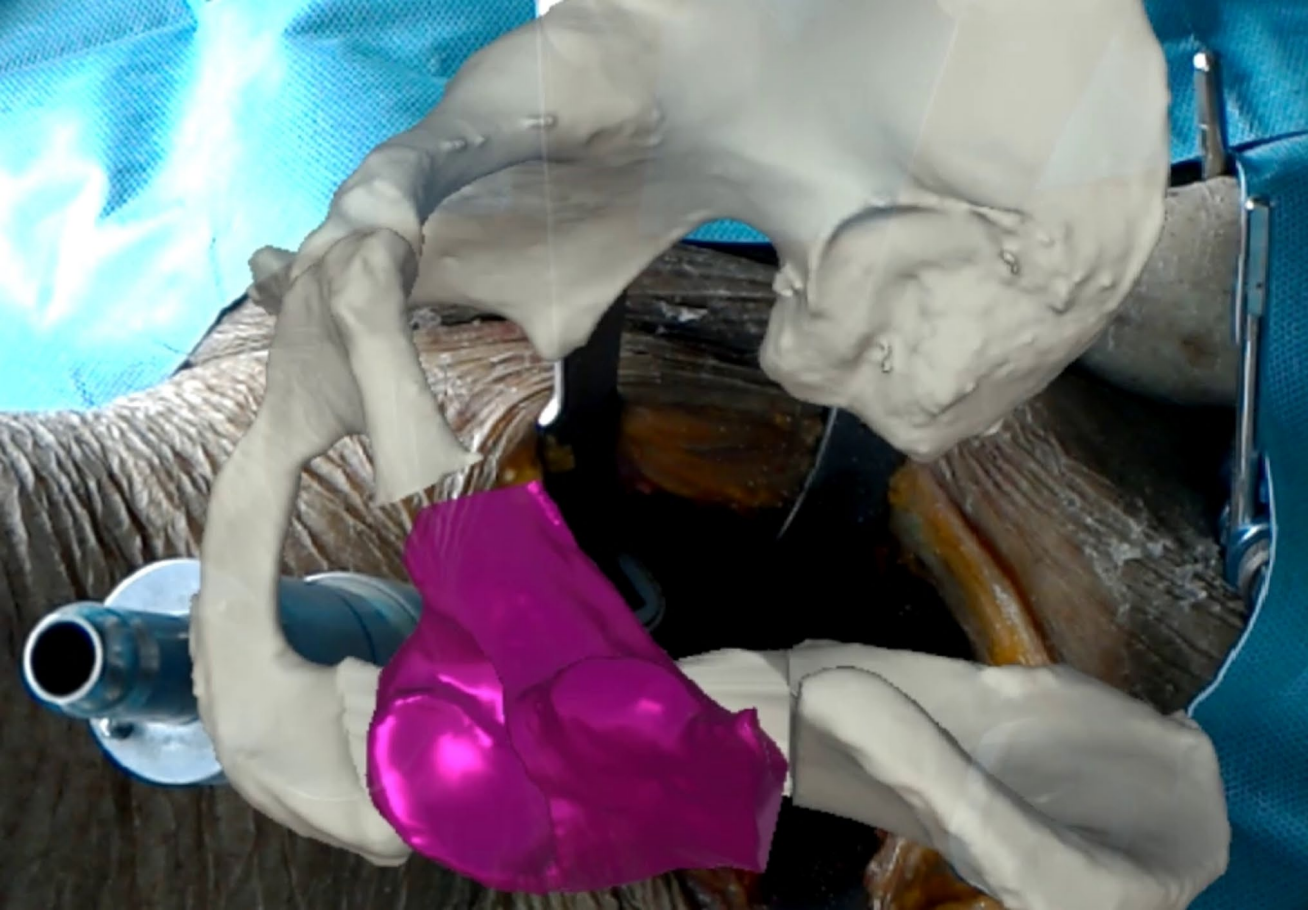
Surgeons intraoperative view with holographic overlay of planned fragment position (purple)

The main goal of our method was to enable the surgeon to perform the osteotomy cuts and reorientation of the acetabular fragment under AR-based surgical navigation. The procedure was executed under realistic OR conditions by two orthopedic surgeons (P.O.Z., S.R.) with extensive experience in hip surgery. Each cadaver was operated on one side by one surgeon and on the other side by the other surgeon to avoid the effect of poor bone quality on the outcome. The standard surgical approach of the PAO [6] was conducted. AR surgical navigation was performed as follows:

1. Surgical approach
  - Skin incision is performed according to Ganz' modified description of the Smith–Peterson approach.
  - Deep dissection is also performed according to Ganz' description but with osteotomy of the anterior superior iliac spine instead of detachment of the tensor fasciae latae and sartorius muscles. The goal was to achieve the usual intraoperative exposure for PAO. For the procedure, the typical surgical instruments were used.
2. Fixation of the markers (Fig. 2a und b)
  The mounts were designed and positioned such that they did not interfere with the surgical procedure nor lead to additional harm on the native anatomy.
  - The pelvic mount was fixed with two screws to the iliac wing near the anteroinferior iliac spine without compromising the future supraacetabular osteotomy. The marker was attached to the mount.
  - The fragment mount was fixed with two screws to the assumed future fragment on the superior surface in order to not compromise the future entry point of the Schanz pin for manipulation of the fragment. The marker was attached to the mount.
3. Registration
  - The previously calibrated HoloLens was put on and the surgeon started the developed application.
  - Definition of 3 predefined anatomical landmarks (Fig. 3) using the PD for coarse registration. These landmarks were selected in a repeatable manner at easily palpable locations (i.e., eminentia iliopubica, spina iliaca anterior inferior, maximal convexity between spina iliaca anterior superior and inferior). The preoperative planning of these points was accessible to the surgeon on a screen, so that he could search for and define the exact same points in the surgical field. This coarse registration served the HoloLens for a rough orientation, so that, for example, errors were avoided, such as a substantial (e.g., 180° or 90°) mispositioning.
  - Digitization of the accessible surface of the pelvis (Fig. 3), namely the area between superior and inferior iliac spine, the ala ossis ilii, the quadrilateral space or the pelvic brim, using the PD for fine registration. After typical exposure of the pelvis, the accessible bone was traced with the PD to collect a point cloud. The marker of the PD was continuously tracked and the HoloLens was enabled to calculate a surface, which was later compared with the surface of the 3D model.
  - Visual verification of registration result based on holographic overlay. If the result was unsatisfactory (> 1 cm estimated offset of the overlay, by eye), the registration process could be repeated until satisfaction. So far, there is no instrument to quantify this satisfaction. Nevertheless, the registration was only accepted if the holographic overlay was congruent in a major part of the visible anatomy or showed deviations of only few millimeters.
  - Registration of the pelvic and fragment markers for later motion compensation and fragment reorientation. For this step, the surgeon had to focus sequentially on either of the two markers and confirm its position upon a satisfactory overlay. For this purpose, a small square was made visible, of which the corners had to correspond exactly with the corners of the markers in the surgical field. In this way, the position of the markers on the anatomy could be assigned exactly to the position of the now registered pelvis. The registration of the markers is reliable, since they are easy for the HoloLens to recognize.
4. AR-based navigation of the osteotomies
  - Navigation of the osteotomies by visualization of osteotomy planes or surgical instruments. Colored semi-transparent osteotomy planes were displayed into the anatomy (Fig. 4a). This enabled the surgeon to align either the saw blade or the chisel with the displayed planes. In addition, the transition point from supra- to retroacetabular osteotomy could be superimposed. All planes could be selected and deselected separately by voice command, so that the surgeon could jump back and forth between different planes to complete them successively, as is the case with conventional PAO. Since there is no direct access to the ischial osteotomy, 3D models of the chisel were displayed (Fig. 4b). The surgeon could align the real chisel with the displayed model to guide this osteotomy.
5. AR-based navigation of the acetabular fragment
  - Reorientation of the mobilized acetabular fragment using a Schanz pin inserted into the supraacetabular bone.
  - Visual verification of the fragment target position based on holographic overlay or the visualized angular deviation between the current and the previously planned fragment target position (Fig. 5). Iterative correction of the fragment position until the desired result is achieved.

After reorientation, the acetabular fragment was fixated to the pelvic bone with screws and the surgical wound was closed layer by layer.

### Step 3—Outcome measurements

Two sets of outcome measurements were obtained. In a first set, the number of registration attempts and total surgery time (skin incision to wound closure) was assessed. In the second set, a 3D analysis was conducted. Therefore, a CT scan of each cadaver after PAO was acquired to obtain 3D bone models. The CASPA software was used to superimpose the pre- and postoperative bone models using ICP [16, 17] and followed by a manual correction of the alignment. The key measures of outcome measurement here were the (a) starting points of the osteotomies, (b) the orientation of the osteotomy planes (supraacetabular and retroacetabular), and (c) the reorientation of the acetabular fragment.

**Fig. 6:**
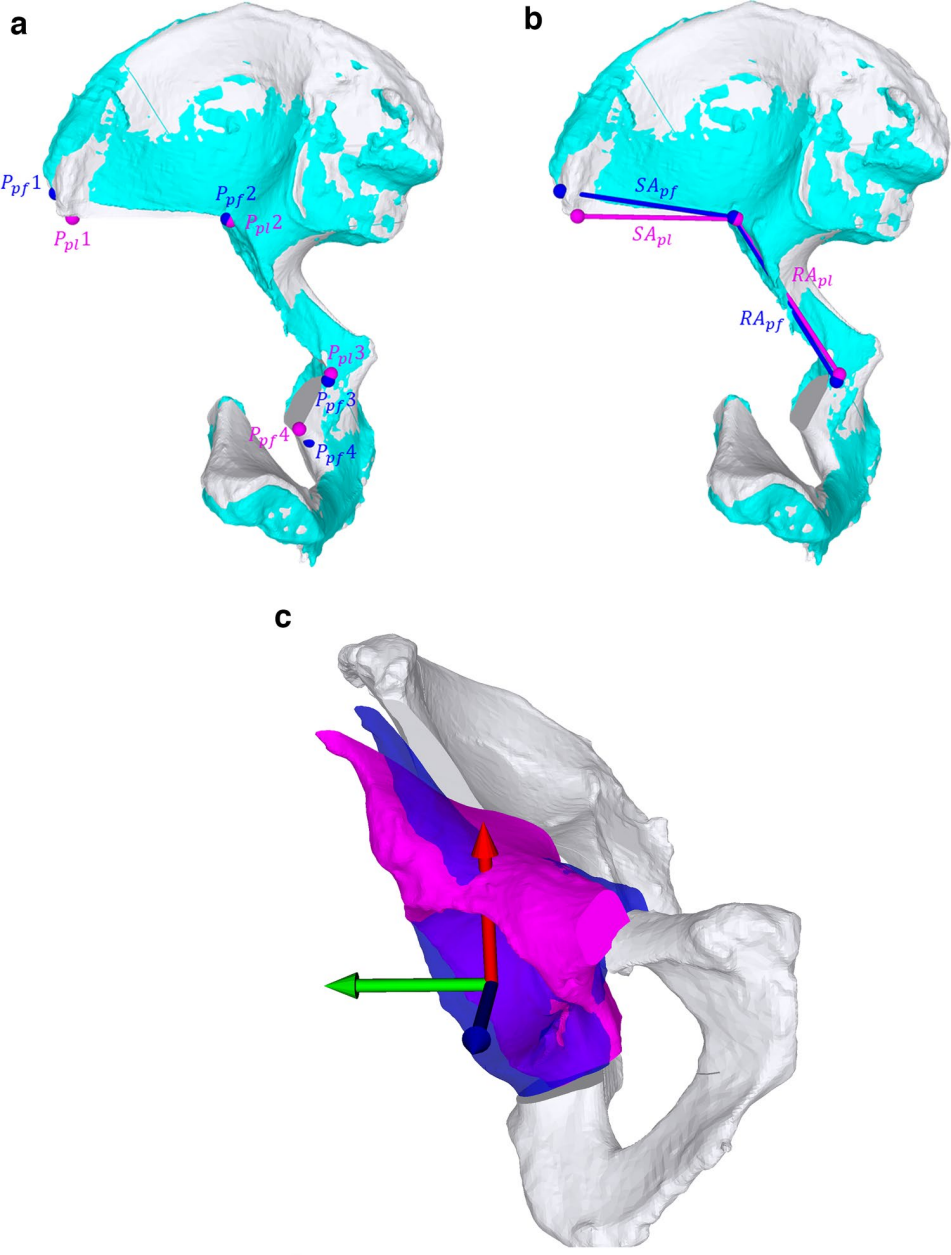
**a** Planned (P_pl_) performed (P_pf_) osteotomy starting points on the bone model pre- (white) and postoperatively (blue). **b** Planned (SA_pl,_ RA_pl_) performed (SA_pf,_ RA_pf_) projected osteotomy planes on the bone model pre- (white) and postoperatively (blue). **c** Planned (magenta) performed (blue) position of acetabular fragment with center of rotation and anatomical planes displayed with a coordinate system (x (red)–y (green)-plane: frontal, x–z(blue)-plane: sagittal) from an anteroposterior view

- Starting points (Fig. 6a).
  The osteotomy starting points were defined as follows:
  Point 1 ('supraacetabular): Most lateral point on intersection between supraacetabular osteotomy plane and corresponding pelvic model.
  Point 2 ('supra—/retroacetabular'): Most superior point on intersecting line between supraacetabular and retroacetabular osteotomy planes.
  Point 3 ('retro- /ischial'): Most superior point on intersecting line between retroacetabular and ischial osteotomy planes.
  Point 4 ('ischial'): Most inferior point on intersection between ischial osteotomy plane and corresponding pelvic model.
  For error quantification the distance [millimeters] was measured between the planned (P_pl_ 1–4) and performed (P_pf_ 1–4) starting points.
- Osteotomy planes (Fig. 6b).
  This measurement was carried out in the form of 2D angles [°] to obtain an illustrative and comprehensible result, which is not the case with 3D angles. Therefore, all points were projected onto a plane defined as the best fit in a least-squares sense by the planned starting points (P_pl_ 1–4). The lines between those projected points were used to quantify the angles between the planned and performed supraacetabular (SA_pl_ and SA_pf_) and retroacetabular (RA_pl_ and RA_pf_) osteotomies. The pubic osteotomy was not quantified because the surface of the osteotomy planes on this very small bone could not be identified with sufficient certainty. The ischial osteotomy was not quantified because it has a curved course (curved chisel) and the definition of a plane was therefore not possible. For both of these osteotomies, the starting point is decisive; this was considered in our assessment (see *Step* 3 a).
- Reorientation of acetabular fragment (Fig. 6c).

A coordinate system was placed in the center of rotation of the hip joint around which the fragment was rotated. This coordinate system was aligned according to the anatomical axes and corresponds to the coordinate system on the basis of which the reorientation was planned. This coordinate system has already been described by Ackermann et al. [1].The reorientation error [°] of the acetabular fragment was measured by calculating the difference of the rotational correction between the planned and the performed position of the acetabular fragment.

Adverse outcomes and serious errors were assessed. Whereas, adverse outcomes described an undesirable outcome with severe consequences due to external circumstances and serious errors described an undesirable outcome with severe consequences due to an incorrect surgical plan or its implementation. The difference is that adverse outcomes cannot be influenced by the surgical technique. An example for this is fractures due to poor bone quality. Serious errors, on the other hand, are caused by incorrect navigation, for example.

## Results

### To what extent can the preoperative three-dimensional plan of this procedure be realized?

#### Outcome set 1 registration attempts

An average of 2 registration attempts (range 1–5) was necessary until a satisfactory registration was achieved. In most of the cases (9 out of 14), the first attempt was satisfactory. The application crashed in 3 cases. Surgery time from skin incision until a stable fixation of the fragment was achieved averaged 56 min. See Table 2.

**Table 2:** Registration attempts and surgery time

Specimen	Side	Registration Attempts	App Crashes	Surgery time [min]*
1	L	1	0	72
	R	2	0	68
2	L	5	1	82
	R	1	0	53
3	L	2	1	51
	R	1	0	54
4	L	1	0	53
	R	3	0	43
5	L	1	0	49
	R	1	0	45
6	L	1	0	43
	R	2	3	69
7	L	1	0	57
	R	1	0	42
Average		1.6	0.4	55.8

*Time from Skin Incision to stable Fixation of Fragment

#### Outcome Set 2 3D analysis

- *Starting points?*
  The mean 3D distance between planned and performed starting points (P_pl_–P_pf_) was 11 mm (range 2–20 mm) for point 1 (supraacetabular), 10 mm (range 3–19 mm) for point 2 (supra- / retroacetabular), 17 mm (range 3–37 mm) for point 3 (retroacetabular / ischial), and 9 mm (range 2–23 mm) for point 4 (ischial), respectively. See Table 3.
- *Osteotomy planes?*
  The mean angle between planned and performed osteotomies was 6° (range 0°–15°) for the supraacetabular and 7° (range 0°–22°) for the retroacetabular osteotomy, respectively. See Table 4.
- *Reorientation of acetabular fragment?*
  The mean reorientation error between the planned and performed rotation of the acetabular fragment was 2° (range 0°–5°) for the x-axis, 11° (range 6°–17°) for the y-axis, and 2° (range 0°–6°) for the z-axis, respectively. See Table 5.

Adverse outcomes

**Table 3:** 3D distance [mm] between planned and performed starting points

ID	1	2	3	4*	5	6	7^+^	8	9	10^+^	11^+^	12	13	14	Mean (Range)
P_pl_–P_pf_ 1	19	18	9	10	7	10	14	11	15	13	8	2	9	8	11 (2–19)
P_pl_–P_pf_ 2	17	8	3	7	3	4	13	7	8	19	8	15	11	10	10 (3–19)
P_pl_–P_pf_ 3	11	17	13	33	35	7	37	9	14	23	20	9	13	3	17 (3–37)
P_pl_–P_pf_ 4	11	2	21	21	23	7	6	13	5	3	4	4	4	4	9 (2–23)

*mm* millimeters, *P*_*pl*_ planned starting point, *P*_*pf*_ performed starting point, *Intraarticular Fracture Runout, ^+^ Fracture of Posterior Column, P_pl_–P_pf_ 1: Supraacetabular starting point, P_pl_–P_pf_ 2: Supra- /Retroacetabular Starting Point, P_p_–P_pf_ 3: Retro- / Ischial Starting Point, P_pl_–P_pf_ 4 Ischial Starting Point

**Table 4:** Angle [°] between planned and performed corresponding osteotomies

ID	1	2	3	4*	5	6	7^+^	8	9	10^+^	11^+^	12	13	14	Mean (Range)
Supraacetabular OT	3	0	6	6	5	7	5	10	11	15	7	2	4	1	6 (0–15)
Retroacetabular OT	0	6	10	19	7	2	12	4	3	22	1	9	1	1	7 (0–22)

*OT* Osteotomy, *Intraarticular Fracture Runout, ^+^ Fracture of Posterior Column

**Table 5:** Reorientation error [°] between planned and performed 3D rotation of the acetabular fragment

ID	1	2	3	4*	5	6	7^+^	8	9	10^+^	11^+^	12	13	14	Mean (Range)
x-axis	1	4	1	5	1	2	1	1	0	2	1	1	2	4	2 (0–5)
y-axis	10	12	7	8	6	10	17	8	8	15	12	14	14	17	11 (6–17)
z-axis	0	1	6	1	4	2	1	2	2	6	3	3	2	1	2 (0–6)

^*^Intraarticular Osteotomy, ^+^Fracture of Posterior Column

In 4 cases, an adverse outcome occurred, which also led to the outliers in the outcome measurement (see Tables 3, 4, 5). These adverse outcomes were an intraarticular extension of a fracture during performance of the retroacetabular osteotomy in one case and a fracture of the posterior column during performance of the ischial osteotomy in three cases. The navigation of the osteotomy planes was followed in these cases and the extension of a fracture occurred independently from the respective planning or its execution.

Surgeons

There was no difference between the two surgeons in terms of accuracy of starting points, osteotomy levels or reorientation of the fragment. The adverse outcomes were all attributable to the same surgeon.

### Is the execution of this complex procedure feasible with AR guidance?

Yes. The cadaver study we performed can be understood as a complex surgical procedure under realistic OR conditions, conducted using AR guidance. The execution of this procedure was technically feasible. In the following, advantages and disadvantages which were revealed during the experiments are reflected:

Advantages of AR guidance: The required equipment (HoloLens, Mounts, Markers) requires only a one-time purchase. The maintenance of this equipment is simple and inexpensive. A cost summary including a comparison to other navigation methods is delivered in Table 6. The required application for intraoperative guidance can be programmed by trained personnel using existing software and is then available for an unlimited number of surgical procedures. There is no need to deviate from the usual surgical procedure; the usual approach must not be extended for the use of compromising devices. Theoretically, there is increased safety in the performance of the surgical procedure through visualization of the hidden anatomy and guidance of the reorientation of the fragment, since the alternative so far has been the inspection with the naked eye, this with a limited insight into the situs. Furthermore, and theoretically when further developed, there is no need for radiological verification during the procedure, which reduces radiation exposure. The planning effort is kept within reasonable limits and can be easily reconciled with the clinical routine.

**Table 6:** Costs

	AR	Freehand	Fl	PSI	OTS
Typ of Navigation/verification	AR/AR	na/radiograph	FI/radiograph	PSI/PSI	OTS/OTS
Costs for aquisition of equipment	4200 USD	na	na	na	na
Costs per case (navigation)	na	na	80 USD	4400 USD	1000 USD
Costs per case (verification)	na	170–340 USD	170–340 USD	na	na
Additional OR time (registration/navigation)	180 USD	na	180 USD	na	180 USD
Additional OR time (verification)	na	360 USD	360 USD	na	na
Additional staff	na	1 Technician for radiographs	1 Technician for radiographs	na	na
Downsides		Radiation exposure, additional staff, time consuming	Radiation exposure, additional staff, time consuming	Expensive, space consuming, larger exposure, occlusion problems	Expensive, space consuming, occlusion problems, off-field tracking

*AR* augmented reality, *Fl* fluoroscopic control, *PSI* patient-specific instruments, *OTS* optical tracking systems, *USD* U.S. Dollar, *na* not applicable

Disadvantages of AR guidance: There is still a certain inaccuracy of the registration and its subsequent display, i.e., overlay of the holographic planning and the reality. In the same instances, the registration process had to be repeated to achieve a satisfactory registration. Until now, the achievement of a satisfactory registration can only be reflected as an impression of the surgeon. The slow speed of the HoloLens processor can lead to delays and, thus, to a prolongation of the operation time. Wearing an OST-HMD during a surgical procedure takes some accustoming and can be uncomfortable for the surgeon. In addition, the device must have been registered as a medical device and intraoperative sterility must not be compromised (see also Table 1). Therefore, a concept was already established, which was settled as a one-time expense.

### What has to be done to proceed to real surgery on patients?

So far, the registration is too inaccurate and not consistent enough which is reflected by the many registration attempts. To improve the registration, some technical deficiencies have to be systematically recorded, quantified and addressed. To quantify the success of the iterative improvement of these deficiencies, a measure for the accuracy of the registration is also required. The accuracy of the OST-HMD must improve to avoid the risk of a surgical procedure being prolonged due to waiting times. Therefore, the comfort and weight of the OST-HMD must also be improved. With 3D navigation, the need to standardize 3D planning is also growing. For this, special planning methods must be developed. Partial automation of planning would then also be conceivable. It would be desirable to carry out the surgical procedure completely without additional steps and also to do it without the installation of markers. For this, an improved registration and a higher speed of processing is necessary.

## Discussion

Periacetabular osteotomy for reorientation of the acetabulum is a complex surgical procedure with limited direct visibility of the acetabulum making execution of the desired correction demanding. Inappropriate osteotomies or an inaccurate reorientation of the acetabular fragment can have adverse outcomes [2, 7, 18]. The current state-of-the-art intraoperative guidance method is fluoroscopy, which comes along with several limitations. A radiation-free and reliable guidance method supporting 3D planning and execution for this inherently three-dimensional procedure is highly desirable. To create the basis for the translation of such a guidance method into the operating room was the designated goal of this work.

This work has some limitations. This investigation was performed on human cadavers without pathological anatomy. Therefore, the correction was based on the correction usually performed. This correction is not reasonable from a medical point of view, but has no influence on the value of our results. The poor bone quality influenced the data in a way that was not in our interest. Nevertheless, we recognized this limitation and this can be considered when interpreting the results.

We were able to perform a complex surgical procedure on cadavers under realistic OR conditions with the AR guidance and without fluoroscopy use.

The mean 3D distance between planned and performed starting points was between 9 and 17 mm, the mean angle between planned and performed osteotomy planes was between 6 and 7 degrees, and finally the mean reorientation error of the acetabular fragment was between 2 and 11 degrees. We consider these values promising regarding the further development of this navigation method, but the interpretation is not trivial. Typically, the malorientation analysis, planning, and verification of the correction in PAO are performed using two-dimensional angles. A comparison of our results with 2D values is tempting but not permissible. In this respect, our results are in accordance with those in the literature regarding 3D evaluation of navigated procedures [11, 14, 20]. Besides the promising results, there were also no obvious serious errors due to poor navigation, whereas the interpretability of the introduced angles is difficult and a definition of what is acceptable has to be defined in the future.. The adverse outcomes are not serious errors resulting from an incorrect surgical plan or an incorrect execution of the surgical steps (i.e., intraarticular osteotomy, osteotomy into the posterior column). Starting points and direction of the osteotomies was conducted correctly in these cases. The fractures occurred from manipulation of the cadaver. This type of manipulation (e.g., extraction of the chisel from an osteotomy) is also performed during state-of-the-art surgery and does not typically lead to a fracture. However, with cadavers, this risk exists due to the poor bone quality. We think that an improvement of accuracy can be reached soon with the emerging technical advancements of the OST-HMDs performance. Furthermore, although the PAO is a delicate intervention, there are still no defined target values and a 3D malorientation analysis and individually tailored planning is lacking. Rather, there is a target range and the accuracy we achieved is able to consider this target range. It would be highly desirable to have a three-dimensional analysis and planning method available for this inherently three-dimensional intervention, which would then form the basis for the individually tailored navigation and performance of the intervention. Compared to other surgical guiding methods (optical tracking systems, patient-specific instruments), AR is inexpensive to purchase and to maintain, as it is reusable. In addition, the same surgical instruments can be used as in the state-of-the-art PAO. The surgeon receives all information in the line-of-sight and does not need screens off-field. Furthermore, no time is lost due to the installation of complex equipment. The registration required a repetition in a minority of cases and in 3 cases the application crashed. The usability is certainly still expandable.

The registration must be improved. The approaches used for registration and tracking are based on general-purpose algorithms. We expect to achieve higher accuracy in the future by including prior knowledge about the anatomy, the procedure and surgical tools. Furthermore, technical deficiencies have to be systematically recorded, quantified, and addressed. To quantify the success of the iterative improvement of these deficiencies, an instrument for the accuracy of the registration is required.

Furthermore, a higher degree of comfort and a higher speed of processing of OST-HMD is necessary to enable a smooth workflow. A higher processing speed could be achieved by implemented streaming solutions. Furthermore, the visualization and, therefore, its perception could be improved in a further step in order to cope with the real patients' anatomy (i.e., colors in good contrast to the red of the blood).

### Conclusion

The augmented reality guidance of the periacetabular osteotomy using an OST-HMD is feasible. We were able to show that the planned correction can be achieved with promising accuracy and without obvious serious errors. Further steps on how a translation from the cadaver to the patient will be possible have been identified and need to be addressed in future work.

### Supplementary Information

Below is the link to the electronic supplementary material.

Supplementary file1 (XLSX 10 KB)
